# Juvenile sialidosis: a rare case and review of the literature

**DOI:** 10.1097/MS9.0000000000001768

**Published:** 2024-02-28

**Authors:** Pashupati Pokharel, Aakriti Dawadi, Biraj Baral, Sunil Dhungana, Arati Baskota, Daman Raj Poudel

**Affiliations:** a Maharajgunj Medical Campus, Institute of Medicine, Tribhuvan University; b Kathmandu Medical College and Teaching Hospital; c Department of Pediatrics, Tribhuvan University Teaching Hospital, Kathmandu Nepal

**Keywords:** case report, mucolipidosis, sialidosis, whole-exome sequencing

## Abstract

**Background:**

Sialidosis is a rare variety of lysosomal storage disease that results in intracellular accumulation of sialic acid containing compounds. The authors report the first case of type II sialidosis, juvenile subtype in a 30-month-old male child from Nepal.

**Case presentation:**

Progressive hearing loss with coarse facies, hepatomegaly, kyphoscoliosis, dysostosis multiplex were the major features in a 30-month-old child born to healthy non-consanguineous parents. With the suspicion of lysosomal storage disease, urinary oligosaccharides were tested and were positive. Whole-exome sequencing revealed a mutation in the neuraminidase gene (NEU1) and established the diagnosis of sialidosis.

**Clinical discussion:**

Sialidosis is a rare autosomal recessive type of lysosomal storage disease resulting due to mutation of the neuraminidase gene leading to intracellular accumulation of sialic acid compounds. Based on the presence of visual symptoms, sialidosis is classified into type I and II varieties. Our case is of type II juvenile sialidosis.

**Conclusion:**

Despite rare, sialidosis is a life-threatening, and disabling disease. Exploring targeted therapy is the utmost to treat this condition.

## Introduction

Highlights

- Sialidosis is a rare autosomal recessive lysosomal storage disease occurring due to mutation in neuraminidase gene (NEU1) located on 6p21.33.
- Mutation leads to intracellular accumulation of sialic acid containing compounds.
- Clinically sialidosis is classified into type I and II variety based on the presence of ocular symptoms.
- Type II sialidosis presents with coarse facial features, visceromegaly, inguinal/umbilical hernia, hearing loss, and dysostosis multiplex.
- Management till date has been limited to symptomatic treatment and management of complications.

Sialidosis, also known as mucolipidosis, is a rare variety of lysosomal storage disorder. It is an autosomal recessive condition occurring due to a mutation in the neuraminidase gene (NEU1) located on chromosome 6p21.33 resulting in deficiency or low activity of the enzyme alpha-N-acetyl neuraminidase and intracellular accumulation of sialic acid containing compounds^1^. The mutation and the amount of functional neuraminidase enzyme closely correlate with the age of onset and the severity of clinical manifestations^2^.

Clinically, sialidosis is classified into type I and II varieties^3^. Patients with type I disease present predominantly with visual symptoms such as blurring of vision, loss of colour vision, night blindness, nystagmus, cherry red macules, and gait abnormalities. Type I variety patients mostly present in the second decade of life, and almost all patients experience myoclonus, ataxia, and seizures as the disease takes its course. However, intellect is generally preserved in type I variety^3^.

Type II can be further grouped into congenital, infantile, or juvenile varieties depending on the age of patient. It could present at birth, known as congenital variety with ascites, oedema, or even hydrops^3^. Furthermore, it may even present after birth, known as infantile or juvenile variety. Patients generally present with coarse facial features, visceromegaly, inguinal/umbilical hernia, hearing loss, and dysostosis multiplex^2,3^. We report the first case of type II sialidosis in a 30-month child from Nepal.

## Case presentation

A 30-month-old male child born to a 32-year-old healthy female was brought by parents to the paediatric outpatient department with complaints of decreased hearing and reduced interaction for 3 months. His parents observed a progressive worsening of his hearing impairment. The child had a normal vaginal delivery at term with no perinatal complications. His developmental history was unremarkable with no neonatal hospitalization and developmental delay. Immunization was completed as per the national schedule. Born to a non-consanguineous marriage, there was no significant family history.

**Figure 1:**
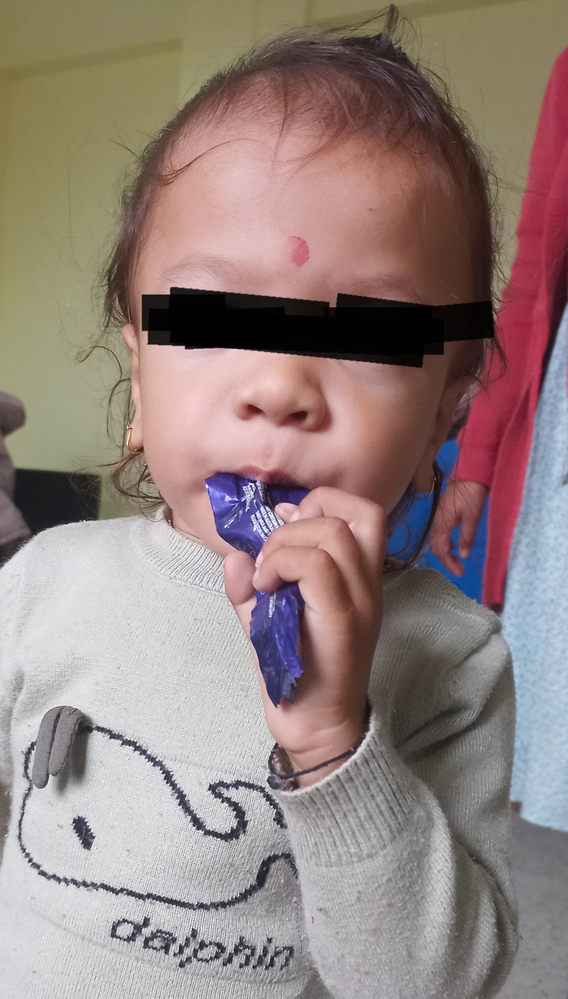
30-month-old child with coarse facies and wide nasal bridge.

**Figure 2:**
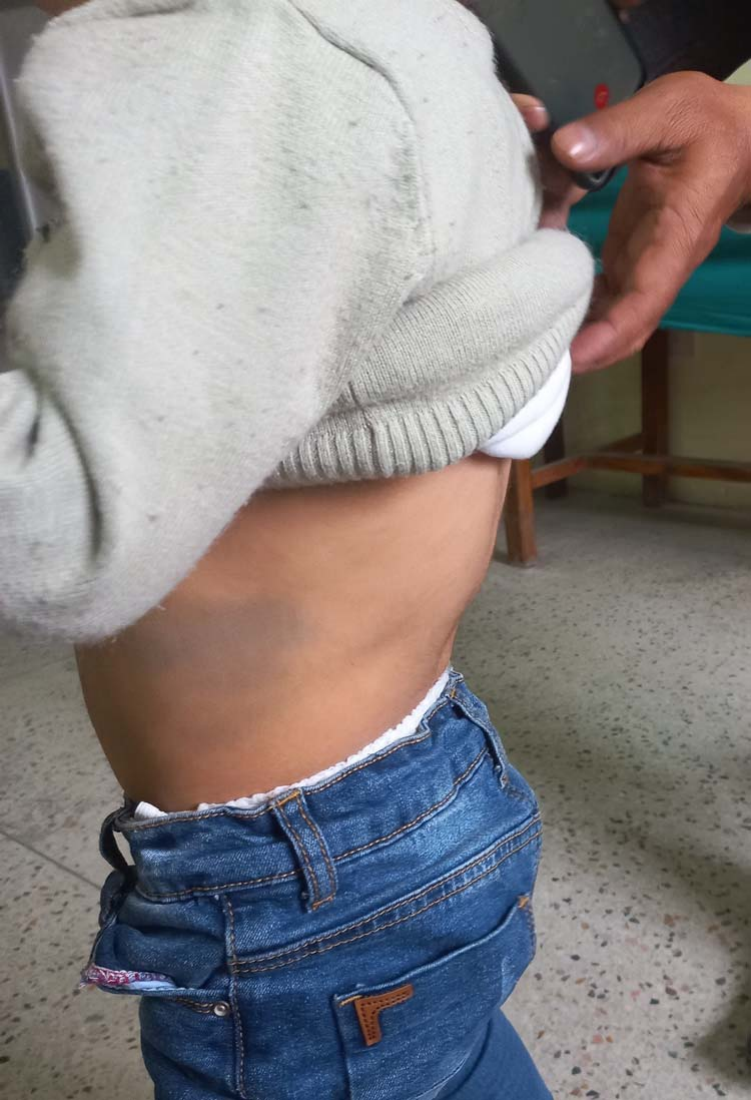
Same child with kyphoscoliosis and a large Mongolian spot on the trunk.

On examination, the baby appeared to have coarse facies with a flat nasal bridge (Fig. 1). He had wide-spaced nipples, a prominent coastal ridge, kyphoscoliosis, and multiple Mongolian spots on the trunk (Fig. 2). Anthropometric evaluation using the WHO chart revealed that the height for age was at zero S.D., weight for age at zero S.D., and head circumference between +1 to +2 S.D. Upon abdominal examination, the liver was palpable 3 cm below the costal margin on the mid-clavicular line. Left lobe was more enlarged than the right lobe. Ocular examination revealed that the child had simple myopic astigmatism. Otoscopic examination of the ear revealed an intact tympanic membrane on both ears. Rest of the systemic examination findings were within normal limits. Based on the history and examination findings, mucopolysaccharidosis, inclusion cell disease, pseudo-Hurler polydystrophy, galactosialidosis, and sialidosis were considered.

Despite ruling out other causes of hearing loss, no conclusive evidence was found. Subsequently, visual reinforcement orientation audiometry was performed which revealed mild hearing loss of 60 dB bilaterally. However, the transient evoked oto-acoustic emission was inconclusive. Thereafter, auditory brainstem response (ABR) was carried out to estimate the hearing threshold. Wave V, being the highest, most stable, and significant, was traceable only up to 70 decibel neural hearing loss in the right and up to 60 decibel neural hearing loss in the left ear. With the impression of bilateral moderate hearing loss based on ABR, the child was advised for a hearing aid trial.

**Figure 3:**
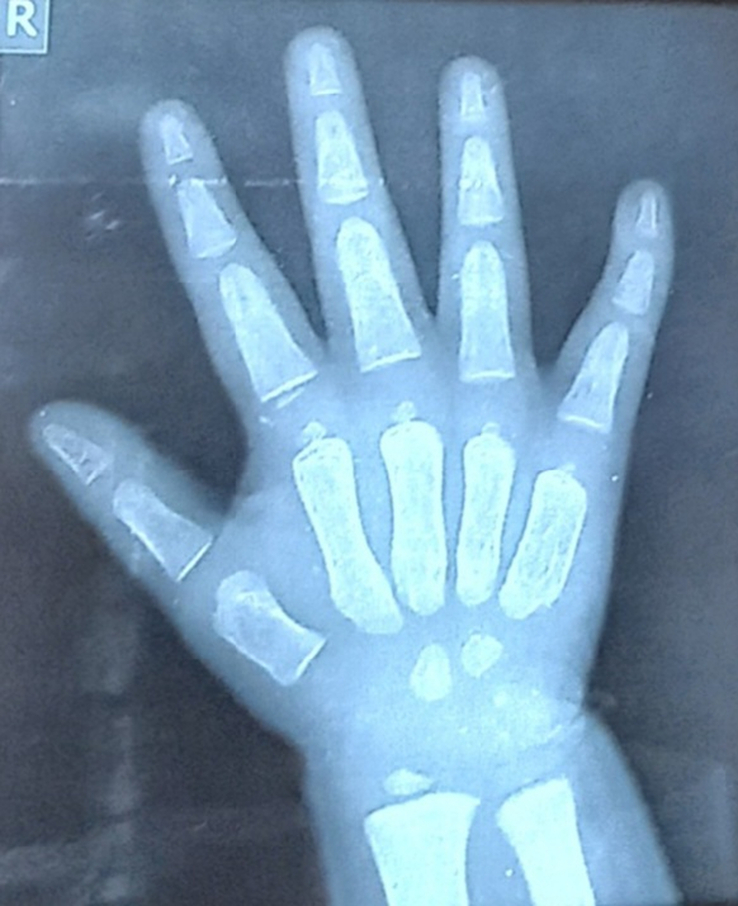
X-ray of right hand with wrist joint showing bullet shaped phalanges.

**Figure 4:**
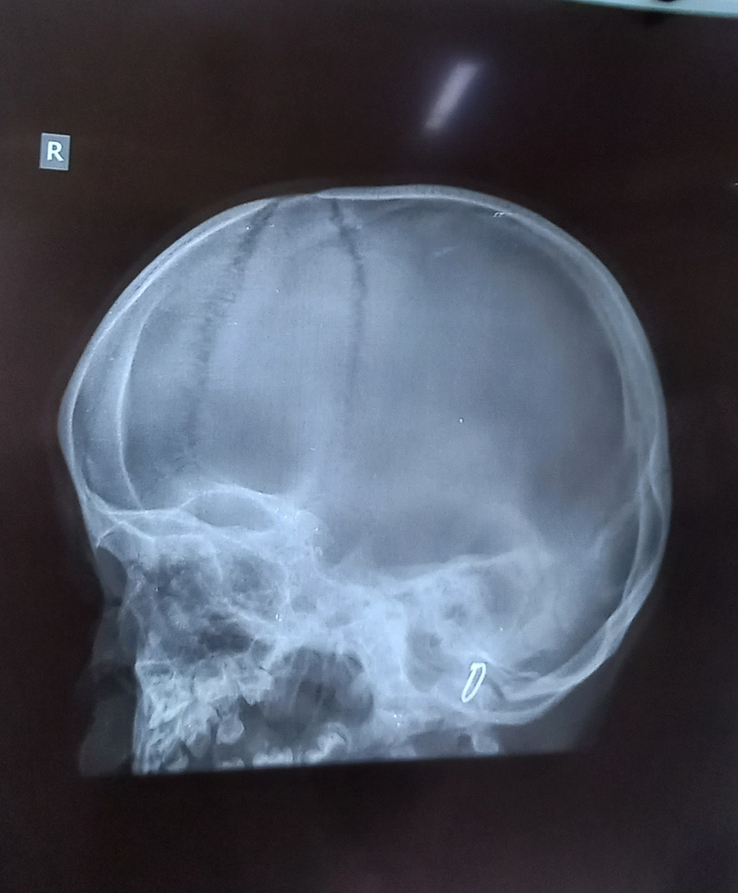
X-ray skull showing sutural diastasis.

**Figure 5:**
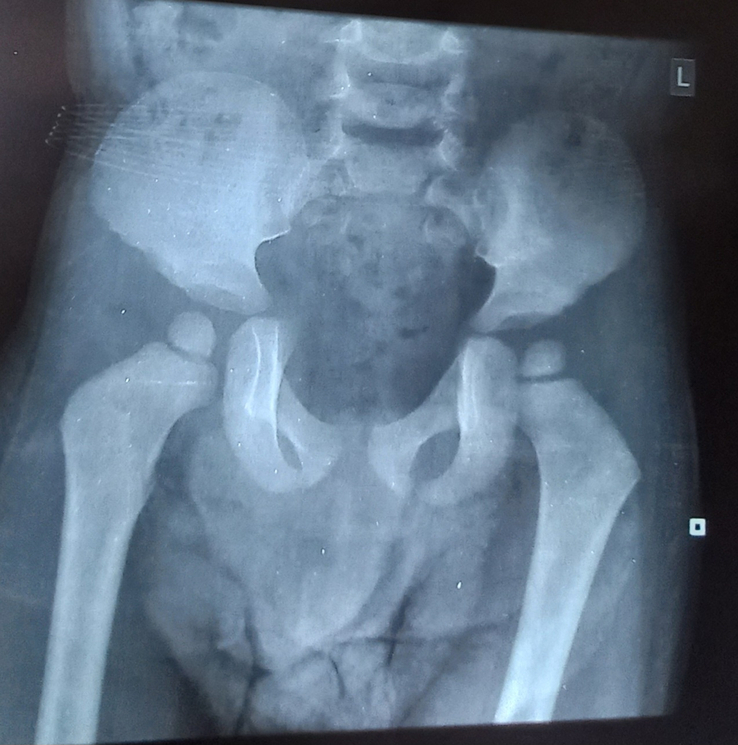
X-ray pelvis with bilateral hip joints showing mild rounding of hip bones bilaterally.

**Figure 6:**
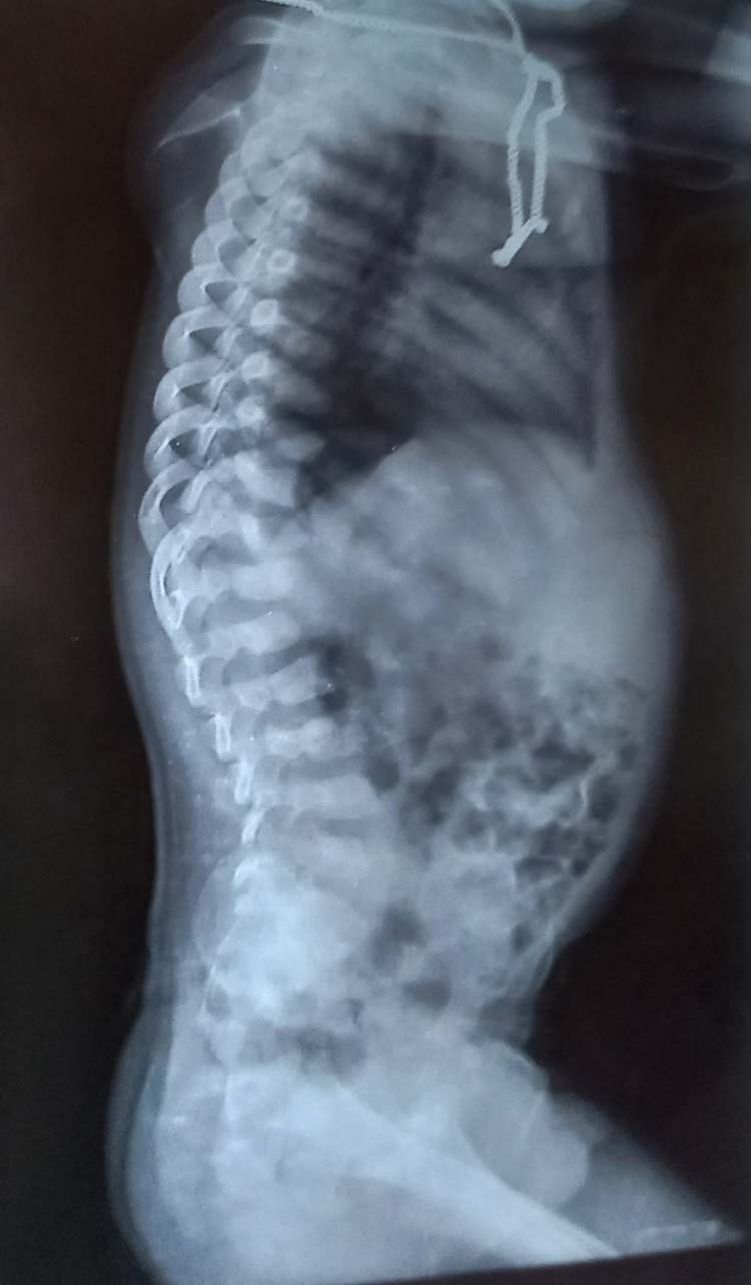
X-ray spine lateral view showing beaking in the thoracolumbar region.

Various investigations were done to further narrow down the differentials. Routine investigations including complete blood count, liver function test, renal function test, electrocardiogram, echocardiography, thyroid function test, and ultrasonography (USG) abdomen were within normal limits. X-ray of the hand, skull, spine, and pelvis were done. In the anteroposterior view X-ray of right-hand bullet shaped phalanges were noted (Fig. 3). On X-ray of the skull, lateral view, sutural diastasis was noted (Fig. 4). X-ray pelvis AP view showed mild rounding of the bilateral iliac bone with normal hip joint bilaterally (Fig. 5). X-ray of the whole spine lateral view showed deficiency of the cortical element in the anterosuperior aspect with beaking in the anteroinferior region in multiple thoracic and lumbar vertebrae. Slight kyphotic deformity was also noted (Fig. 6). These radiological features were suggestive of mucopolysaccharidosis.

Based on this, urinary glycosaminoglycans (GAG) were tested but the results were negative. Additionally, enzyme assays for iduronate-2-sulphate sulphatase, alpha-L iduronidase, arylsulphatase-A, hexosaminidase-A all came within normal limits. Furthermore, enzyme assays done on a blood sample for alpha-Mannosidase and alpha-fucosidase, showed normal enzyme activity. Finally, urinary oligosaccharide analysis was done which came out to be positive.

Whole-exome sequencing revealed a homozygous missense variant in Exon 4 of NEU1 gene at codon 227. This is a proven pathognomic variant of sialidosis and this resulted in amino acid substitution of arginine for glycine. Although the diagnosis of sialidosis was made, optimum therapeutic measures are yet to be identified. So, the patient was advised for symptomatic treatment of hearing and eyesight problems and visited for regular follow-up.

## Discussion

We report the clinical picture of a rare lysosomal storage disorder, juvenile sialidosis type II. To the best of our knowledge, this is the first case of sialidosis in Nepal. According to estimates, sialidosis (types 1 and 2 combined) affects between 1/5 000 000 to 1/1 500 000 live births annually^2,3^. Only 19 occurrences of the infantile/juvenile variant of sialidosis II were found globally between 1980 and February 2018^4^.

Dysmorphic features are noted to be present in all cases of infantile/juvenile sialidosis II but absent in all cases of type I^5^. This formed the basis for our case to be categorized as a case of type II sialidosis. The most concerning issue that required a hospital visit in our instance was a progressive loss of hearing. As suggested by a study in mice, hearing loss occurs due to the involvement of both conductive and sensorineural components^6^. In a 1987 study that organized the data of 50 sialidosis patients, only 1 out of every 15 children with type II juvenile sialidosis exhibited hearing loss, whereas another study from 1980 to 2018 and found that 10 out of 19 individuals experienced hearing loss^4,5^. In general, progressive hearing loss is correlated with the disease progression^4^. However, in our case, hearing loss was the primary concern, suggesting a potential delay in suspicion and diagnosis of the patient.

Ophthalmological findings such as cherry red spot, although present in almost all cases of type I and less than 75% cases of type II, were absent in our case except a simple myopic astigmatism^7^. Developmental delay has been a major reason for seeking medical attention, as reported by Arora and colleagues, Ranganath and colleagues^8,9^. However, to date our patient has attained all his milestones as appropriate for his age. His overall intelligence appears appropriate as well, exhibiting characteristics of Sialidosis type I, in which intelligence is almost always retained^3^. However, the age of onset, lack of myoclonus, and ophthalmological findings distinguish it from a type I cases.

Skeletal abnormalities present in our patient, mainly kyphosis, dysostosis multiplex, were somewhat similar to that of a case of mucopolysaccharidosis^10^. This led to a diagnostic dilemma which was eliminated when the urinary GAG levels came out to be negative.

Seven cases as reported from northern India shared a missense variant of the mutation (c.679G > A; p.Gly227Arg)^8^. Whereas, other cases reported from the southern part of India showed other forms of mutation^11,12^. Our case shares the same mutation as those cases reported from northern India. This similarity observed could suggest a common geographical origin of the mutation, which would be difficult to know since the missense variety of mutation is a common type of mutation observed in different parts of the world^13^.

Dilated cardiomyopathy has been reported in a case of sialidosis II in a newborn who died at 11 months of age^14^. Similarly, a report of left ventricular enlargement with mild to moderate decrease in cardiac function has been reported^15^. In the same article, it was noted that a delay in the onset of symptoms correlates with a decreased likelihood of cardiac involvement. As our patient had no evidences of cardiac involvement, thus, it is rare for him to have any cardiac involvement in the future.

Nephrosialidosis, a rare manifestation of sialidosis II, has been reported in only 16 cases. The age of onset was between 2 and 3 years of age, almost all patients had hepatomegaly^16^. Our patient, falling in the above-mentioned age range, also having hepatomegaly, whether he will develop nephrosialidosis is a matter of concern and should be assessed for the same in follow-up.

Treatment options for neuraminidase deficiency have been limited to mouse models^17,18^. The specific treatment for sialidosis in humans and its outcome still require a long dedication. Furthermore, establishing specific treatment for sialidosis II patients with shorter life expectancy is still a long way. Maximum patients of sialidosis II were observed to be moribund with death occurring in around the second decade of life^4^. Our patient has been receiving symptomatic treatment in order to mitigate the difficulties in daily life. He has been provided with a hearing aid for hearing disability, glasses for eyesight problem, and surgery is advised if kyphosis becomes debilitating.

The only limitation or challenge faced while writing the case was not being able to diagnose the condition within Nepal due to lack of genetic tests. Furthermore, despite diagnosis lack of definitive treatment strategies is even a great challenge for sialidosis patients.

## Conclusion

Sialidosis, with its autosomal recessive pattern of inheritance, underscores the importance of efforts to minimize consanguinity through genetic counselling and the identification of heterogeneous individuals to mitigate the impact of this disease. Furthermore, targeted therapies for sialidosis need to be explored by the scientific community.

## Ethical approval

Not applicable.

## Consent

Written informed consent was obtained from the patient’s parents for publication and any accompanying images. A copy of the written consent is available for review by the Editor-in-Chief of this journal on request.

## Source of funding

Not applicable.

## Author contribution

Conceptualization: P.P. Writing—original draft: P.P., A.D., B.B. Writing—review and editing: P.P., A.B., S.D., D.R.P. Visualization and supervision: P.P., D.R.P. All authors have read and agreed to the final version of the manuscript.

## Conflicts of interest disclosure

Not applicable.

## Research registration unique identifying number (UIN)

Not applicable.

## Guarantor

Pashupati Pokharel.

## Data availability statement

Not applicable.

## Provenance and peer review

Double anonymized.
